# Antimicrobial Activity of a Vaginal Gel Formulation: Considerations Related to Vaginal Infection and Dysbiosis

**DOI:** 10.3390/pathogens10121576

**Published:** 2021-12-03

**Authors:** Francesco De Seta, Bryan Larsen

**Affiliations:** 1 Department of Medical Sciences, University of Trieste, 34149 Trieste, Italy; francesco.deseta@burlo.trieste.it; 2 Institute for Maternal and Child Health IRCCS “Burlo Garofolo”, 34137 Trieste, Italy; 3 College of Osteopathic Medicine, Marian University, 3200 Cold Spring Road, Indianapolis, IN 46222, USA

**Keywords:** vaginal infection, dysbiosis, topical treatments, antibacterial, anti-fungal, EDTA, non-prescription formulation

## Abstract

Many non-prescription preparations intended to treat or alleviate symptoms of vaginal infection are available in American and European markets, but many have scant preclinical or clinical research underpinning. Respecta^®^Balance Gel (RBG) is marketed as an adjunct to probiotic treatment and its relevant antimicrobial properties were studied. Key findings with the manufacturer-supplied gel showed reduced turbidity in broth-dilution tests by 50% against *Candida albicans* and *Candida glabrata* at RBG concentrations 0.2–0.4% of neat product, respectively. A 50% reduction in turbidity of *Escherichia coli*, *Streptococcus agalactiae*, *Enterococcus faecalis* ranged from 1.6–2.2% and *Gardnerella vaginalis* was shown by flow cytometry counts to undergo a 50% reduction at 0.3% RBG. Propidium iodide staining indicated a rapid reduction of cell integrity of *G. vaginalis* almost immediately while after 4 h 45% of *E. coli* cells were stained. The lactic acid in BHI inhibited bacteria and yeast at concentrations ranging from 0.2–1.8% but inhibition was not solely due to pH since a 1:4 dilution of RBG resulted in a pH near neutral (6.75). Other findings showed biofilm accumulation assessed after 10-days exposure of *Candida* spp. to RBG and was reduced by an average of one-third (community strains) to one-half (drug-resistant strains). One excipient of the RBG, disodium EDTA, inhibited the growth of bacteria and yeast at concentrations below those present in RBG and may accentuate the activity of the host defense factor, lactoferrin. We conclude that RBG is a potent inhibitor of vaginal microorganisms relevant to vaginitis or intrapartum infections and contains excipients that may contribute to its antimicrobial activity.

## 1. Introduction

For decades infectious diseases have been addressed largely through the application of antibiotics to specific conditions. In the case of gynecologic and obstetrical infections, antibiotics have served both for therapy and prophylaxis. This primary reliance on antibiotics engendered antibiotic guidelines and standards of care that inform medical practice worldwide [1,2,3]. In addition to antibiotics, however, allowances are made for use of non-antibiotic modalities in some situations. Concern over indiscriminate and excessive use of antibiotics has given rise to the concept of antibiotic stewardship that promotes diligent and consistently rational use of these compounds [4,5] but is largely implemented in hospitals. Antibiotic stewardship in ambulatory settings seems to have growing adherents [6,7] with emphasis on using antibiotics only when essential. While antibiotics will remain the mainstay of therapy, non-antibiotic treatment modalities could provide useful alternatives that circumvent selective pressure for antibiotic resistance and supply non-prescription treatments for infectious conditions that do not demand physician-directed intervention [8,9,10].

Infections encountered in obstetrics and gynecology have generated more scientific interest contemporaneously with a better understanding of the microbiome of the lower female genital tract [11,12,13] and elucidation of microecological interactions between the indigenous microbiota and common vaginal infections such as bacterial vaginosis (BV) and vulvo-vaginal candidiasis (VVC) have resulted. 

Women with vaginal symptoms often turn to over-the-counter (OTC) preparations, nutraceuticals, and supplements or non-prescription medical devices to treat conditions that may or may not be due to the most common infectious causes including VVC [14] or BV [8,15]. The practice is controversial as physicians worry about self-misdiagnosis, developing antifungal resistance, or off-target effects [14]. Patients pursue non-pharmaceutical preparations in a desire for convenience and autonomy in managing their condition as evidenced by the robust sales of such products. In a recent article by Tidbury et al [16], the current state of non-antibiotic treatments was summarized with emphasis on probiotic organisms and lactic acid, sucrose, and estriol in relationship to BV. Numerous natural compounds have been proposed for VVC [17,18] and some have even been evaluated in clinical trials [19]. Practitioner concerns notwithstanding, the widespread use of non-prescription health care products will likely continue, suggesting that evaluation (including laboratory and clinical studies) of such products, available direct to the consumer, is warranted.

In this paper, we present studies performed to determine if a proprietary topical product for vaginal health, Respecta^®^ Balance Gel (RBG) produced by Giellepi SpA, Milan Italy, has demonstrable biological activities appropriate for its intended use as an adjuvant in treating or preventing symptomatic vaginal conditions [20]. This gel formulation is balanced with lactic acid (specific concentration not stated by manufacturer) to an acidic pH (3.5 to 4.0) and includes other components with possible antimicrobial activity such as EDTA, and as studied previously by our group, farnesol [21]. The formulation also incorporates prebiotics (polydextrose, glycogen, xanthan gum) and excipients to establish appropriate physical properties such as Carbopol and PEG-40. While recognizing that excipients in addition to those with potential antimicrobial activity can influence biological effects, this paper will focus on laboratory findings in relation to a range of microbial species, emphasizing *Candida* spp. and *Gardnerella vaginalis*.

## 2. Results

**Figure 1:**
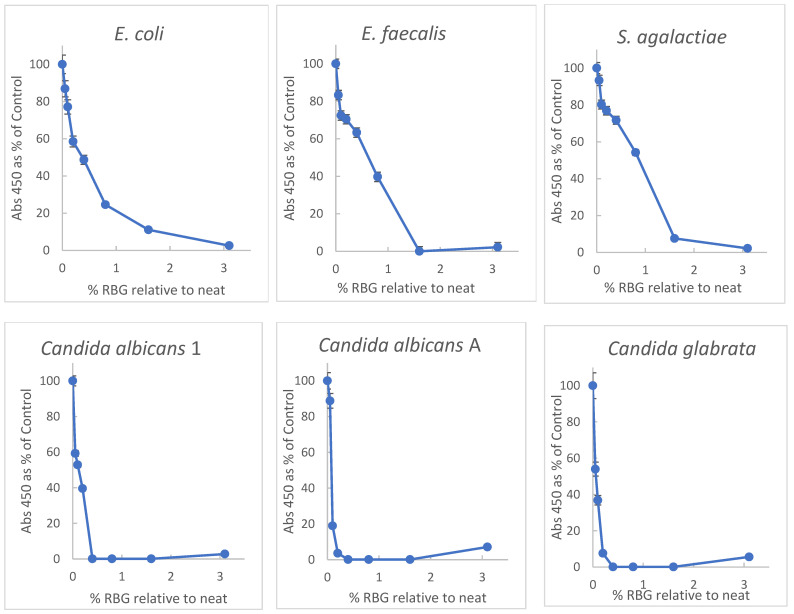
Inhibition of yeast and bacteria by RBG (Respecta Balance Gel) as percent of growth in BHI growth medium. RBG was diluted in BHI and concentrations of RBG from 50–0.1% relative to neat RBG in triplicate. Concentrations on *x*-axis represent fractional concentration of neat RBG; *y* axis values represent the mean of 3 values for OD450 change after 18 h incubation. Relative standard deviations indicated by error bars ranged from 0.2 to 7%. All test organisms were ATCC strains except *Candida albicans* 1, which was a community isolate. See Methods for additional information on the test organisms.

Primary among properties of proposed therapeutics for vaginal infections or dysbiosis is the ability to inhibit growth or induce damage to relevant microbial species. Initially, doubling dilutions of RBG in brain-heart infusion broth (BHI) were inoculated with a multi-taxon set of organisms including non-fastidious bacteria and yeast for broth-dilution growth measurement. Turbidity was measured at 450 nm in each well before and after overnight incubation at 37 °C. Because the baseline turbidity of the undiluted gel was high, practical evaluation of turbidity was possible at dilutions ≥ 1:32. Plating 10 μL aliquots from dilutions from 1:2 through 1:32 revealed no growth for any of the test organisms (sensitivity 1 × 10^2^ cfu/mL). Optical density (OD) readings were normalized with growth in BHI representing 100% to facilitate interspecies comparison with dose-response data plotted in Figure 1.

Figure 1 shows a broad inhibitory activity toward all the tested organisms, and interestingly the yeast strains appeared more susceptible to the RBG effect. Based on the highest dilution with OD ≥ 0.2 after incubation, *Candida albicans* were inhibited by 0.2%, *Candida glabrata* by 0.4%, and bacterial isolates by 1.6% for *E*. *coli* and *Enterococcus faecalis* and 2.2% for *Streptococcus agalactiae*. From the data, the 50% effective dose (concentration relative to neat RBG) was estimated. ED50, based on the data in Figure 1 furnished ED50 concentration values of RBG as follows: *E. coli*, 0.34% RBG; *E. faecalis*, 0.56%; *S. agalactiae*, 0.9%; *C. albicans* 1, 0.12%; *C. albicans* A, 0.08% and *C. glabrata*, 0.03%.

**Figure 2:**
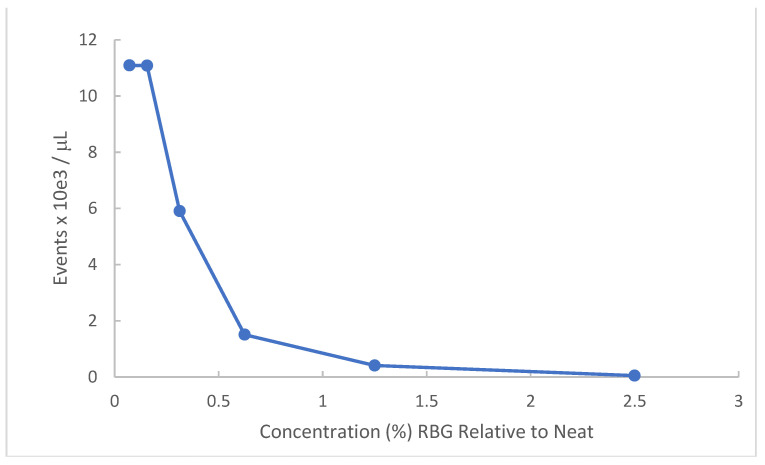
Effect of RBG on *G. vaginalis* strain AMD. *Gardnerella vaginalis* strain AMD from V-agar was inoculated into dilutions of RBG in BHI. After overnight incubation at 37 °C in 5% CO_2_ atmosphere, each dilution was applied to the Accuri C6 flow cytometer, reported as events/μL (*y* axis) using forward scatter channel events. RBG expressed as % relative to neat is displayed on the *x* axis.

*Gardnerella vaginalis* has particular relevance to symptomatic vaginal conditions. This organism is not in and of itself the single cause of bacterial vaginosis (BV) but is present in most symptomatic occurrences and when in abundance represents one of the sentinel organisms for BV. Because the organism is fastidious and of relatively small size compared to other bacteria, turbidity measures are less successful. Accordingly, we employed flow cytometry to obtain a dose-response of RBG to this organism. Figure 2 provides data for a *Gardnerella vaginalis* strain AMD (see methods). Bacteria recovered from V-agar plates were incubated with dilutions of RBG overnight at 37 °C in 5% CO_2_ and resulting cultures were counted by flow cytometry.

As indicated by this experiment, *Gardnerella vaginalis* strain AMD appears highly susceptible to the antibacterial activity of RBG. The lowest dilutions representing 2.5% and 1.25% RBG showed complete inhibition of *Gardnerella vaginalis* strain AMD. The ED50 was estimated as 0.34% RBG. 

**Figure 3:**
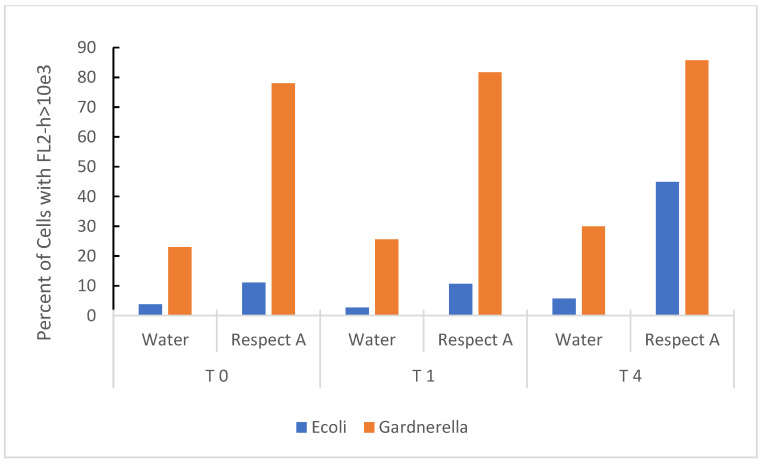
Time-based effect of RBG on bacterial organisms at 2.5% RBG relative to neat. *E. coli* from overnight culture in BHI and *Gardnerella vaginalis* strain AMD from V-agar were placed in sterile nanopure water or 2.5% RBG (relative to neat), stained with PI and fluorescence measured (*y* axis) at timed intervals (in hours, *x* axis). Bacterial cells were identified in the forward scatter channel and fluorescence channel 2 (threshold for staining was set at FL2-h based on heat-inactivated bacterial cells).

Propidium iodide (PI) staining, a marker of non-viability, was used to indicate damage to the bacterial membrane. Figure 3 shows the cumulative effect of RBG expressed as a percent of bacteria taking up PI over 4 h. Both *E*. *coli* and *Gardnerella vaginalis* strain AMD underwent membrane damage upon exposure to the RBG, and the effect was almost instantaneous for *Gardnerella vaginalis* (Time 0 actually provided for a brief time lag from the addition of bacteria, mixing in the test medium, and loading the samples onto the flow cytometer). *E. coli* was also included as a less fastidious comparison organism, and it also increased PI staining over 4 h exposure to 2.5% RBG. *Gardnerella vaginalis* strain AMD appeared more susceptible to this treatment compared to *E. coli*, but *E. coli* despite being more tolerant of RBG, increased in propidium staining over the time course of the experiment.

**Figure 4:**
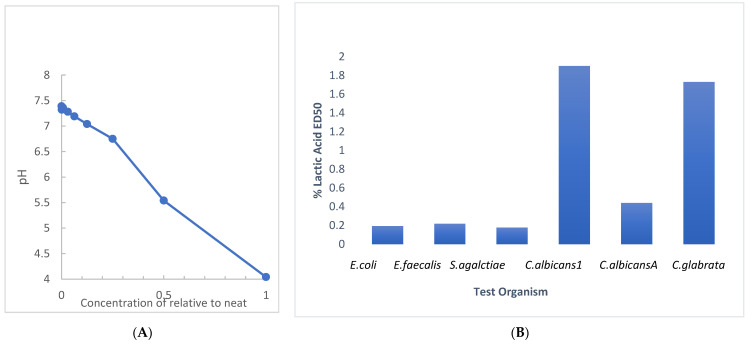
Evaluation of dilution and lactic acid on pH and antimicrobial effect. (**A**) The pH of RBG as supplied by the manufacturer measured 4.04. The pH of serial two-fold dilutions of RBG in BHI were tested (*y* axis). At a 1:64 dilution, RBG pH (7.32) was nearly the same as BHI (7.39). (**B**) Serial dilutions beginning at 5% lactic acid were prepared in BHI and were inoculated as in Figure 1 and ED50 were estimated from turbidity measurements.

RBG is manufactured to a pH between 3.5 and 4 (manufacturer information) [20] with a sufficient volume of 80% lactic acid to attain the target pH. Since we evaluated the antimicrobial activity of RBG with a broth dilution technique, we evaluated the effect of dilution on the pH of RBG in BHI, with the results shown in Figure 4A. A near-neutral pH of 6.75 was obtained at a 1:4 dilution (25% relative to neat). This undermines an assumption that pH is the sole source of RBG antimicrobial activity. The assessment of the antimicrobial effect of lactic acid in BHI was performed with the multi-taxon set used in Figure 1 and indicated that ED50 values for four of six test organisms (Figure 4B) showed inhibition under conditions where pH would be above 5. The same test organisms were also mixed into RBG adjusted to 7.0 with 0.1 N NaOH and 10 μL samples were sub-cultured using a loop onto BHI agar. When no growth was seen after 24 h the plates were held for an additional 7 days, and no growth was observed from either the neat or pH adjusted RBG. Taken together, these pH-related studies suggested that RBG has antibacterial activity due to components in addition to lactic acid and its pH effect.

EDTA is one of the excipients in RBG that may contribute to antimicrobial activity, and preliminary evidence of lactic acid-EDTA interaction was sought. The multi-taxon set of test organisms was challenged with broth dilutions of 0.2% w:v disodium EDTA in BHI, lactic acid at 5% v:v, and dilutions of a combination of equal parts of 0.2% EDTA and 5% lactic acid and turbidity was measured after overnight incubation. As in previous experiments, ED50 for individual and combination dilutions was evaluated. Table 1 summarizes the results which, although limited to a single data set, indicate ED50 values tended lower for combinations, but simple isobolograms using these data suggested neutral or additive effects for the three bacterial strains and antagonism for the three yeast strains. However, since all dilutions of the combination had the same ratio of lactic acid to EDTA, a more comprehensive experiment with checkerboard assay would be warranted in future work.

**Figure 5:**
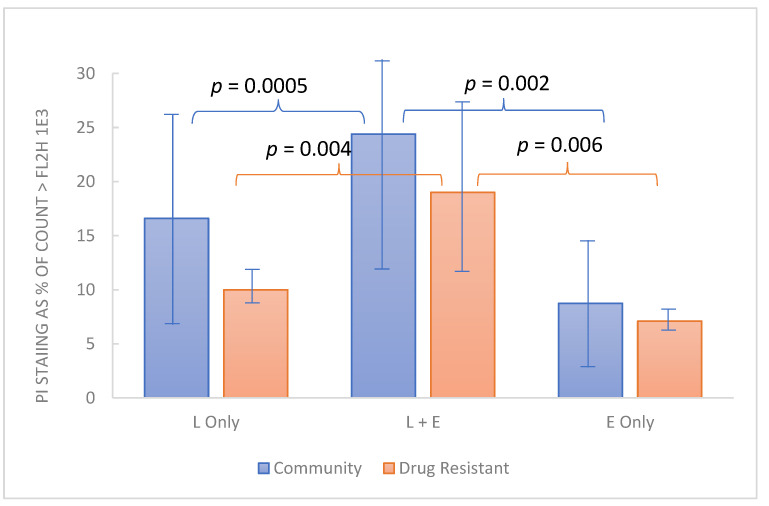
Combined effect of lactoferrin and EDTA on cell integrity among yeast strains. Test organisms grown overnight in BHI at 37 °C were dispensed into 1% bovine lactoferrin, 0.1% EDTA or both, each (1% lactic acid with 0.1% EDTA) and incubated for 1 h. Propidium iodide staining (*y* axis) represents the % of organisms stained (inactivated). Each bar represents the average PI staining for 12 *Candia* strains from the community-acquired organisms or the drug-resistant organisms from the ATCC MP8 panel. Normal distribution of data among various strains was supported by Kolmogorov-Smirnov computation and comparisons were made by paired t test with *p* < 0.05 considered significant.

Of additional interest is the possibility that in clinical use, EDTA may enhance the action of host defense factors. Accordingly, preliminary evaluation of EDTA in relation to lactoferrin was undertaken using crystalline bovine lactoferrin (see Methods). The test organisms used for this experiment included 12 drug-resistant *Candida* strains from the MP8 panel and 12 non-drug-resistant community strains. Because this experiment employed 1 h exposure at 37 °C, involved a single concentration of the test articles (1% w:v lactoferrin and 0.1% disodium EDTA in BHI final concentration), and derived results by flow cytometry, it was feasible to use a larger number of fungal strains. PI staining was employed to determine the viability of the test organisms. Each organism was dispensed in order so analysis could be performed in a “first-in-first-out manner”. As indicated by the data (Figure 5), in the aggregate, the non-drug-resistant organisms appeared slightly more robust than the drug-resistant group, but all organisms were demonstrated to be more susceptible to cell integrity compromised in the presence of lactoferrin and EDTA compared to either, individually.

Biofilm plays an important role in mucosal infections and specifically, in this present context, vulvovaginal candidiasis, and bacterial vaginosis. Therefore, preliminary information on biofilm generation in the presence of RBG was evaluated using strains of *Candida* (drug-resistant and non-resistant community strains) for in vitro biofilm production and assessment of inter-strain variation. All test organisms were grown in BHI to prepare an inoculum (100 μL directly dispensed from the starter culture) for the experiment. Bacterial isolates were not evaluated. Biofilm was developed in the presence of 2.5% in half-strength BHI and compared to half-strength BHI by safranin staining of adherent biomass (see Methods for procedural detail). Since biofilm involves stress responses, it was decided to incubate well plates for 10 days (37 °C, 5% CO_2_). Control levels of biofilm for each strain were determined in half-strength BHI and compared to the same strain incubated with 2.5% RBG in half-strength BHI. Because half of the test organisms were drug-resistant and the other half from community isolates, these were evaluated as separate groups with the results presented in Table 2. For all strains tested, the biofilm estimated by safranin stain was diminished in all cases by incubation with RBG, however, the range was large and some strains showed very small decreases in biofilm, others were more substantially affected. The differences in RBG effect on biofilm between drug-resistant and community isolates were not significant. In the aggregate, diminution in biofilm relative to controls was on average reduced by one-third to one-half. Because this study was limited in addressing many of the variables of concern to a thorough investigation, it serves mainly to suggest that a more detailed understanding of the effect of RBG on biofilm should be sought.

## 3. Discussion

The research presented here evaluated the antimicrobial activity of a non-prescription, substance-based topical medical device, RBG, which the manufacturer describes [20] as “adjuvant in the treatment of vaginitis and vaginosis specifically studied for rebalancing the physiological pH and vaginal microbiota”. This product combines several ingredients (see Methods) relevant to vaginal conditions other than vaginitis (such as pruritis, odor, or dryness), but the foremost characteristic supporting RBG use for vaginal infections is antimicrobial activity. Selecting several organisms representing yeast (*Candida albicans* and *Candida glabrata*,) and bacteria (including Gram-negative and positive organisms) for testing we did a broth-dilution experiment with turbidity as an indicator of growth. Because of the viscosity of the formulated product, it was initially diluted on a w:v and because of its optical density of dilutions < 1:32 were too high to allow meaningful turbidity measures therefore plating (10 μL) on BHI agar confirmed a lack of growth in lower dilutions (see methods). Internal validity for this experiment was supported by the OD of each well being measured before and after incubation with all conditions set up in triplicate. OD as a percent of controls is plotted in Figure 1 and dilutions ≥ 1:32 and reveals inhibition of all test organisms in a dose-dependent manner. It might be noted that *Candida albicans* A is multi-resistant to antifungal drugs. Interestingly, growth inhibition by RBG was greater for yeast isolates than bacteria (Figure 1).

The initial demonstration of antimicrobial activity against our multi-taxon panel (bacteria and yeast) was limited to six organisms, and additional microbial species relevant to vaginal infection would be useful in future work. Lactic acid-based products other than RBG have been tested against *Chlamydia*, gonococcus, and Human Herpes Virus [22,23] which were outside the scope of this work. We did, in a separate experiment, demonstrate that a strain of *Gardnerella vaginalis* was also inhibited by RGB. Due to its fastidious growth requirements, we found it advantageous to use bacterial counts made by flow cytometry to demonstrate this organism was not detected in 1.25% RBG (relative to neat). The literature is replete with studies showing lactic acid bacteria having an inimical effect on *Gardnerella vaginalis* which is not derived solely from lactic acid. Less information is available on the direct effect of lactic acid on *Gardnerella vaginalis*, although the maximum concentration of lactic acid produced in cultured *Lactobacillus* was 2.5 mg/mL [24] and a reported minimal inhibitory concentration for lactic acid was 3.6 mg/mL against a single strain of *Gardnerella vaginalis* [25]. 

While larger test organism panels might discover strains or species resistant to RBG, the limited panel of organisms suggested that RBG diluted to 5% or 10% would be expected (based on subculture experiments) to inhibit all test panel organisms. Applying this information to intravaginal use, we estimate that a 3–5 mL vaginal application of RBG would result in a dilution of approximately 1:2 [26] and would be expected to retain an antimicrobial effect, at least at the time of application. Such a prediction requires in vivo confirmation and demonstration of the persistence of the product in situ. In addition, the work of O’Hanlon et al [27] indicates that many in vitro studies do not consider the presence of endogenous lactic acid or the anaerobic conditions under which vaginal bacteria exist, which may imply lactic acid containing vaginal products may have different potency in vivo than would be predicted from in vitro studies. Our incubation conditions did not employ an anaerobic atmosphere and suggests an opportunity for future work.

Initial growth studies were augmented by a limited experiment in which RBG diluted 1:40 (or 2.5% relative to the neat formulation), compromised membrane integrity of *E. coli* and *Gardnerella vaginalis.* In this time-based experiment (Figure 3), rapid intrusion of propidium iodide into *Gardnerella* was observed but occurred more slowly with *E. coli*. Although the literature on lactic acid in compromising microbial membrane integrity is limited, a study on *E. coli* 0157:H7 showed 1% lactic acid to be effective in causing solute leakage from membranes [28]. However, based on the estimated concentration of lactic acid in a forty-fold dilution, the lactic acid would probably be insufficient by itself to induce membrane damage. This leads to consideration of other components of RBG that may contribute to its overall antimicrobial effect. Further, lactic acid is harmful to bacteria only in its protonated state [29], so we determined how RBG dilution affects pH, especially since there are no known buffers in the RBG formulation. While neat RBG had a pH of 4.04 (Figure 4) where less than half of the lactic acid would be protonated, dilution raised pH and at 12.5 % RBG to pH 7, yet that concentration of RBG (Figure 1) was inhibitory to all organisms tested, again suggesting additional components of RBG should be considered as contributing to RBG antimicrobial activity.

Microbiologic observations on lactic acid appear commonly in the food science literature but fewer in vitro studies are related to BV associated bacteria [27], *Neisseria gonorrhea* [30], *Chlamydia trachomatis* [31], or Herpes virus [32] and one report indicated that *Candida albicans* and *Candida glabrata* inhibition was more dependent on acetic than lactic acid [33]. Perhaps because it is generally recognized as safe (GRAS), lactic acid formulations have commonly been included in vaginal products that have been studied clinically, but any new formulations (even if based on presumably safe components) would benefit from preclinical in vitro studies as presented here.

A systematic review of the literature regarding the use of lactic acid products for BV and microbiota regulation [34] reported that among three studies comparing lactic acid to metronidazole, one study reported equality and two studies reported inferiority of lactic acid. Despite the relatively large number of articles examined, they reported on only 7 products with suitable data to analyze. Interestingly, the doses of lactic acid in the products varied dramatically with concentrations in the applied product ranging from 0.06% to 3.9%, and for products having total dose listed, ranging from 40 mg to 700 mg (as oligomeric lactic acid for the latter). One of the products did not list a concentration or dose and one contained lactic acid and sodium lactate. All products had pH in the range of 3.5–4.5, like RBG (pH 3.5–4), although the RBG manufacturer does not state the exact lactic acid concentration. Variations among products included a variety of excipients and were also superimposed on variability in study design, and the likelihood of bias in evaluated clinical studies was noted in the review [34]. The vagaries of the clinical studies noted and the variety of formulations, underscores the importance of considering excipients delivered along with lactic acid in the many products studied clinically. The work of Moench et al [23] is particularly instructive regarding the biological effects of excipients.

Since RBG contains EDTA which may contribute to antimicrobial activity, we showed EDTA at concentrations present in RBG inhibited six test organisms (Table 1) and the ED50 values for EDTA alone ranged from 2.6% to 9.5% of the amount present in neat RBG. When combined with lactic acid in a ratio considered like that in RBG, inhibition was maintained but we were unable to support additive or synergistic activity through isobole calculation. Only by checkerboard assay would a definitive conclusion be made regarding the synergistic activity. EDTA is commonly reported to be a synergist for various antimicrobials [35,36,37,38] and should be more comprehensively evaluated in future work.

In addition to augmenting antimicrobial agents, EDTA has also been reported to enhance the activity of host defense factors including lysozyme and lactoferrin, [39] both of which are found in cervical secretions [40,41]. Preliminary evidence for the augmentation of lactoferrin by EDTA was demonstrated in this paper (Figure 5). Having established that RBG can cause membrane compromise, we used flow cytometry to measure PI staining of 24 yeast isolates treated with EDTA, lactoferrin, or both. After 1 h between 10–25 percent of yeast cells had taken up propidium iodide. For both yeast panels, a larger percentage were stained when lactoferrin and EDTA were combined, although each compound individually had an effect on membrane integrity. It will be interesting to further study this from a mechanistic standpoint as it is possible that the iron-chelating activity of lactoferrin may be augmented by that of the EDTA. Earlier reports also indicated that lactic acid permeabilizes the outer membrane of Gram-negative organisms through chelation and is as effective as EDTA and potentiates the effect of lysozyme [42]. In the context of RBG which contains both lactic acid and EDTA, the interactions deserve more detailed evaluation particularly in the context of host factors. An incidental finding was the drug-resistant strains appeared slightly less affected by EDTA and lactoferrin, but the mechanisms involved were not explored in this study.

Biofilm research has grown in scope and complexity in recent years and the preliminary, and admittedly, basic effort to see if RBG may reduce biofilm was useful mainly as a first step to the subsequent investigation. Biofilm technologies, both in terms of methods and specialized apparatus are impressive, but subject to scrutiny as numerous variables in organisms, media, growth conditions have limited the ability to compare results between studies [43]. A simple exploratory experiment was used to obtain preliminary information on *Candida* biofilm in the presence of a single concentration (2.5%) of RBG against 24 *Candida albicans* strains (12 community isolates and 12 drug-resistant isolates described in Methods). In terms of incubation time, the times reported in the literature differ widely, but one report which observed biofilms over 140 days, referred to 2–12 days to be short term incubation [44] while, for practical reasons including accelerating data collection, short incubation times are often employed. Differing also from other research, we did not replenish the medium during the incubation period based on the role microbial stress response and persister formation have a role in biofilm generation [45]. Undoubtedly, a more sophisticated, and detailed investigation including the additional collection of interim data and manipulation of nutritional support, should follow as biofilm suppression represents an important potential attribute of RBG if confirmed.

Because we demonstrated that RBG has antimicrobial activity, it is possible biofilm may have been blocked by the initial antimicrobial effect, although the presence of small amounts of biofilm would argue against this. It is possible to dissect the biofilm development with more detailed experiments. For example, while lactic acid and EDTA may prevent initial growth, farnesol, a component of RBG is a quorum-sensing molecule and may play a role in decreased biofilm formation [21]. While farnesol may diminish hyphal growth and biofilm, the generation of quiescent persister cells may enhance the survival of *Candida albicans* [45]. Another excipient not addressed by this study was hydroxyacetophenone, which was not anticipated to contribute significantly to antimicrobial activity, but in the complete RBG formulation may add to overall activity [46]. Likewise, the presence of mucoadhesive compounds in RBG could provide surface interactions that interfere with the adherence of microorganisms to abiotic substrates. A separate study of interactions between these various excipients would be warranted in the future both for RBG as well as other complex consumer products.

While the goal of demonstrating antibacterial activity of RBG and its components was foremost in our research, the intent of the manufacturer to use RBG as an adjunctive treatment to rebalance the vaginal microbiota remains a problem for future research. In addition to in vitro studies of interactions among individual components of RBG a study of the possible antimicrobial effect of RBG on lactobacillus indigenous to the vagina or used as probiotics should be undertaken and in a clinical investigation of the effect of RBG on vaginal lactobacillus pursued. Such studies would round out the findings reported here.

## 4. Materials and Methods

### 4.1. Respecta^®^ Balance Gel (RBG) 

The formulated product was shipped directly from Giellepi, SpA (Milan, Italy), to our laboratory in Indianapolis (IN, USA) and was immediately placed at 4 °C prior to use. Upon arrival, samples of RBG were applied with a sterile swab to blood agar plates incubated at 37 °C in 5% CO_2_ confirming the absence of detectable microorganisms in the material provided. RBG was viscous and adherent to volumetric equipment and had a milky appearance, requiring some initial steps before use, detailed below (Section 4.4). 

Visible cloudiness of dilutions remained up to a 1:32 dilution which obviated the use of turbidity readings for the dilutions below 1:32. However, all dilutions from 1:2–1:1028 were added to microtiter wells and inoculated with test organism, but OD readings below 1:32 were not used. Instead, 10 μL samples were spotted on BHI agar to determine the presence (>1 × 10^2^ cfu/mL) of viable organisms.

Information supplied by Giellepi SpA (Milan, Italy) indicated that the formulated RBG contained in addition to water, disodium EDTA, polydextrose, glycogen, xanthan gum, sodium hyaluronate, propylene glycol, farnesol, decylene glycol, hydroxyacetophenone, PEG-40, hydrogenated castor oil, tocopherol acetate, and lactic acid (sufficient 80% lactic acid) to achieve a final pH of 3.5–4. The exact quantities are proprietary information, but we are authorized to state the concentration of EDTA is 0.2% which informed experiments with EDTA.

### 4.2. Reagents and Chemicals 

Blood agar plates were obtained from BD BBL (Cockeysville, MD, USA) for general testing of sterility of RBG and V-agar (BD BBL) was used for propagation of *Gardnerella vaginalis*. BHI agar was made by adding 1.5% bacteriologic grade agar before autoclaving and pouring into plates.

Bacteria and yeast were grown in BHI broth (DifCo Laboratories, Detroit, MI, USA) which was prepared and autoclaved according to manufacturer instructions. BHI was also used for the dilution of RBG. 

Lactic acid 85% and disodium EDTA were ACS grade. Propidium iodide (Millipore Sigma, St. Louis, MO, USA) was made as a 100 mg/mL stock solution and added to samples to be stained for flow cytometry at 10 μL/mL of the sample.

Crystalline bovine lactoferrin was supplied by Giellepi SpA (Milan, Italy), dissolved in sterile water at a concentration of 2%, and stored at 4 °C until use. 

### 4.3. Test Organisms 

For this research, 31 test organisms, described below, were available as frozen (−80 °C) stocks that prior to use, were allowed to thaw and inoculated into BHI, grown overnight at 37 °C before further use, except *Gardnerella vaginalis* strain AMD. *Gardnerella vaginalis* was obtained from BEI Resources (Manassas, VA, USA) and sub-cultured from frozen stock directly onto V agar and grown for 48 h at 37 °C in 5% CO_2_ and colonies lifted directly from the plate and sub-cultured again on V agar and colonies were picked and suspended into BHI for immediate use.

Scouting general antimicrobial activity employed a multi-taxon selection of organisms that included 3 bacterial and three fungal species. Bacteria were *E. coli* ATCC 11775, *Streptococcus agalactiae* ATCC 13813, and *Enterococcus faecalis* ATCC 19433. The three fungal strains included one *Candida albicans* (abbreviated CA 1) obtained from a local clinical laboratory as further explained below. The other two strains were *Candida glabrata* ATCC 2001 and *Candida albicans* ATCC 64124 selected from the ATCC MP8™ multi-drug resistance pattern of resistance to 3 echinocandins, 3 triazoles and intermediate resistance to 5-flucytosine. For convenience, this organism is abbreviated here as CA A.

For experiments that employed a range of *Candida* isolates, a set of organisms which included 12 community isolates (abbreviated CA 1 mentioned in the multi-taxon set above was included with 11 others designated as CA 2-12). The clinical isolates, provided by a local clinical laboratory, had been isolated and characterized by standard methods and supplied to us without patient information. Drug-resistant *Candida albicans* were from the ATCC MP8™ panel included the following: ATCC 64124 (mentioned above as part of the multi-taxon set), ATCC 10231, ATCC 76485, ATCC 28121, ATCC 90819, ATCC MYA-1023, ATCC MYA-427, ATCC MYA-574, ATCC 38289, ATCC 11651, ATCC 96901, ATCC 90029 (all trademarked and referred for convenience as CA A–L, respectively). Additional information including the antifungal drug resistance pattern infection site and the geographic location of origin for each strain are available on the ATCC product information documents (ATCC.org).

### 4.4. Microbiologic Methods

To accommodate the rheologic and optical properties of RBG the following steps were taken before its use in experiments. Due to its viscosity, RBG was not readily measured with volumetric equipment and was first diluted 1:2 with sterile water by aseptic transfer to pre-weighed sterile tubes, re-weighed and an equal weight of sterile water added. After vigorous mixing, further dilutions were made into a brain-heart infusion (BHI) medium with volumetric equipment. In the dilution process, care was taken to thoroughly mix (exhaustively pipetting up and down) each dilution before transferring to the next dilution blank. For each organism tested, a total of 11 serial two-fold dilutions, beginning with 1:2 RBG in BHI were dispensed in 200 μL volumes in 96 well plates and an equal volume of BHI in the 12th well served as the growth control. Sufficient wells were prepared to allow triplicate determinations for each of the six test organisms in the multi-taxon set. Starter cultures of test organisms were prepared by a subculture of growing cultures overnight in fresh BHI and turbid starter cultures were diluted 100-fold in sterile water and 10 μL of this initial dilution was used to inoculate wells of test plates. Prior use of these organisms indicated that this inoculum procedure provided 1 × 10^4^–1 × 10^5^ organisms/mL. After setting up the plates, OD was measured at 450 nm to establish baseline readings for each well. The same procedure was used for measuring the antimicrobial potency of lactic acid and EDTA.

Low dilutions of RBG had high background turbidity which precluded the use of OD readings for dilutions < 1:32 consequently, after overnight incubation, 10 μL aliquots from each well were spotted on BHI agar. The absence of growth on BHI agar indicated fewer than 1 × 10^2^ viable organisms/mL were present. Turbidity was documented by a microplate reader with a 450 nm filter. The starting OD for each well was subtracted from the corresponding OD after incubation and the change was reported as the ratio of growth in the treated well to the growth in BHI controls. The 50% effective dose (ED50) was used to indicate the concentration of inhibitor at 50% of the difference between maximum growth (BHI) control and starting absorbance of the culture. It was calculated by linear interpolation of the concentration of inhibitor using OD values below 50% and above 50%.

Measurement of OD at 450 nm has been used previously by us and although higher wavelengths are more commonly used, precedence in the literature for this wavelength exists [47,48,49,50,51] and is claimed more sensitive at lower cfu numbers [49] where inflection of the dose-response curve begins. Both 450 nm and 700 nm show OD proportional to cfu [51] and given prior reliance on the lower wavelength in our laboratory it was continued for these studies. *E. coli* growth in rich medium produces a strong signal at 450 nm with OD 1 = 4.09 × 10^9^ cfu/mL [50]. Considering the pre and post-incubation measures of OD, we considered that observations made at 450 nm had internal validity.

Flow cytometry provides a precise measure of microbial cells and when coupled with propidium iodide staining, also permits estimation of the proportion of cells that are damaged. Our laboratory uses the Accuri C6 cytometer which features a peristaltic pump sample injection which allows accurate quantitation of events/μL, unlike pneumatically applied samples. However, we chose to limit the use of flow cytometry to those experiments where propidium iodide staining was the endpoint, experiments with *Gardnerella vaginalis*, which provided little turbidity in BHI, and where minimal amounts of RBG (which contains mucoadhesive components) would contact the flow cell to reduce the risk of fouling the flow cell. Propidium iodide 10 μL (from 100 mg/mL stock) was added 10 min before applying the sample to the cytometer. After the addition of the propidium iodide, samples were placed in the dark at room temperature (drawer below the cytometer) and applied one-by-one to the instrument. Scatter plots of FSC-h versus FL2-h with the FL2 marker set at FL2-h = 1 × 10^3^ provided the number and percent of stained and unstained events. The fluorescence threshold was established previously by comparing heated to unheated propidium iodide-stained bacterial samples.

While a variety of sophisticated methods and apparatus are employed in biofilm studies, we intended to pursue a simple polystyrene plate method [51] to determine if RBG might affect biofilm development. Both sets of yeast (see 4.3 above) representing 12 community isolates (Strains CA 1–CA 12) of *Candida albicans* and 12 antifungal drug-resistant strains from the ATCC MP8 were grown overnight in BHI broth. Biofilm estimates were based on 2 mL cultures prepared in 24 well plates with diluted RBG and compared to wells with BHI control medium. Cultures were held for 10 days at 37 °C, and the plates remained undisturbed; refeeding did not occur. After incubation, the contents of each well were aspirated off, washed three times with water to remove planktonic organisms, and stained with 1% safranin for 1 h at room temperature. The unbound stain was aspirated off and wells were again washed three times with water and allowed to dry. The stain remaining in the wells represents the adherent biofilm and the color was mobilized into reagent alcohol and read spectrophotometrically at 560 nm. Absorbance due to biofilm in test wells was divided by absorbance in control (BHI) wells and reported as percent of control.

### 4.5. Statistical Analysis 

In addition to descriptive statistics (mean and standard deviation), comparison of means by paired or unpaired t-test were calculated using Microsoft XL. Significance was set at *p* < 0.05. Where needed, evidence of normal distribution of data was supported by measures of skew and kurtosis as well as Kolmogorov-Smirnov calculations.

## 5. Conclusions

RBG is a formulation of components generally recognized as safe in the marketed formulation. This research proved RBG inhibitory toward vaginal microorganisms relevant to vaginal infection, dysbiosis and prematurity. Data were provided that showed the activity of lactic acid and EDTA contribute to the antimicrobial activity. The work presented indicates a robust antimicrobial effect of RBG toward bacteria including *Gardnerella vaginalis* and *Candida albicans* that resists the effect of dilution that would be expected on intravaginal application. Antimicrobial activity is provided by lactic acid, although our results suggest that additional components of the RBG formulation may contribute to antimicrobial activity including EDTA. Bacterial and yeast membrane integrity was compromised by exposure to RBG, and artificial biofilm generation was diminished. The findings of possible interactions among components of RBG, though limited, may be valuable in hypothesis generation for additional mechanistic or possible interactions that may occur when RBG encounters vaginal fluid. Based on demonstrated antimicrobial effects, further clinical evaluations seem appropriate, particularly to determine if RBG can restore a healthy vaginal microbiome in women affected by dysbiotic vaginal conditions and if it affects probiotic lactobacilli.

## Figures and Tables

**Table 1:** Broth dilution demonstration of antibacterial effect of lactic acid, EDTA alone and in combination.

Test Organism	ED50 for Lactic Acid (% v:v)	ED50 for Disodium EDTA (% w:v)	ED50 for Combination (% Lactic Acid/% EDTA)
*E. coli*	0.192	0.0059	0.09/0.0036
*E. faecalis*	0.217	0.0191	0.204/0.0082
*S. agalactiae*	0.176	0.0101	0.132/0.0025
*C. albicans* strain 1	1.90	0.0058	0.565/0.0226
*C. albicans* strain A	0.44	0.0052	0.141/0.0056
*C. glabrata*	1.73	0.0053	0.452/0.0108

Serial two-fold dilutions of 5% lactic acid, 0.2% disodium EDTA, and a combination of lactic acid and EDTA in a 25:1 ratio in BHI were inoculated with the test organisms as in prior experiments. After overnight incubation at 37 °C, OD450 was measured and used to estimate ED50 as in earlier experiments.

**Table 2:** Biofilm produced by *Candida albicans* strains A-L and *Candida albicans* strains 1–12 in the presence of RBG at 2.5% relative to neat.

	Range (Biofilm as % of BHI Control)	Average (Biofilm as % of BHI Control) ± SD	Average % Biofilm Reduction
Community/non-resistant N = 12	15.7–91.8	61.5 ± 22.5	38.2
Drug resistant strains N = 12	19.3–64.3	48.6 ± 13.8	51.4

Biofilm was established in 2 mL volumes containing 1 mL BHI plus 1 mL of 5%RBG with respect to neat (final concentration of RBG was 2.5%) and inoculated with each of 24 *Candida* strains. 12 strains were from the ATCC MP8 drug resistant panel and 12 were community strains (see Methods section). Incubation time was 10 days without change of media or refeeding. Relative biofilm quantity is: (Abs 560 from RBG treated well)/(Abs 560 from control culture) × 100. Comparison of means was by unpaired, two tailed t test *p* = 0.105.

## Data Availability

Not applicable.

## References

[1] Mendling W., Brasch J., Cornely O.A., Effendy I., Friese K., Ginter-Hanselmayer G., Hof H., Mayser P., Mylonas I., Ruhnke M. (2015). Guideline: Vulvovaginal candidosis (AWMF 015/072), S2k (excluding chronic mucocutaneous candidosis). Mycoses.

[2] American College of Obstetrics and Gynecology (2020). ACOG Practice Bulletin 215. Vaginitis in Non-Pregnant Patients. https://www.acog.org/clinical/clinical-guidance/practice-bulletin/articles/2020/01/vaginitis-in-nonpregnant-patients.

[3] Centers for Disease Control and Prevention Sexually Transmitted Infections Treatment Guidelines 2021. Morbidity Mortality Weekly Reports Reommendations and Reports 70. https://www.cdc.gov/std/treatment-guidelines/STI-Guidelines-2021.pdf.

[4] Dyar O.J., Huttner B., Schouten J., Pulcini C. (2017). ESGAP (ESCMID Study Group for Antimicrobial Stewardship). What is antimicrobial stewardship?. Clin. Microbiol. Infect..

[5] Brotherton A.L. (2018). Metrics of Antimicrobial Stewardship Programs. Med. Clin. N. Am..

[6] Rowe T.A., Linder J.A. (2019). Novel approaches to decrease inappropriate ambulatory antibiotic use. Expert Rev. Anti-Infect. Ther..

[7] Keller S.C., Cosgrove S.E. (2020). Reducing antibiotic resistance through antibiotic stewardship in the ambulatory setting. Lancet Infect. Dis..

[8] Vodstrcil L., Hocking J., Law M., Walker S., Tabrizi S.N., Fairley C.K., Bradshaw C.S. (2013). Hormonal Contraception Is Associated with a Reduced Risk of Bacterial Vaginosis: A Systematic Review and Meta-Analysis. PLoS ONE.

[9] Milandri R., Bocchialini T., Maltagliati M., Michele C., Simonetti E., Stefania F., Maestroni U.V., Rocco B.M.C., Micali S. (2020). Effects of D-Mannose, Ellirose^TM^ and *Lactobacillus plantarum* in treatment of urinary tract recurrent infections (rUTIs): A survey of urologists knowledge about its clinical application. Acta Biomed..

[10] Batoni G., Maisetta G., Esin S. (2021). Therapeutic Potential of Antimicrobial Peptides in Polymicrobial Biofilm-Associated Infections. Int. J. Mol. Sci..

[11] Kalia N., Singh J., Kaur M. (2020). Microbiota in vaginal health and pathogenesis of recurrent vulvovaginal infections: A critical review. Ann. Clin. Microbiol. Antimicrob..

[12] Muzny C.A., Łaniewski P., Schwebke J.R., Herbst-Kralovetz M.M. (2020). Host–vaginal microbiota interactions in the pathogenesis of bacterial vaginosis. Curr. Opin. Infect. Dis..

[13] Ceccarani C., Foschi C., Parolin C., D’Antuono A., Gaspari V., Consolandi C., Laghi L., Camboni T., Vitali B., Severgnini M. (2019). Diversity of vaginal microbiome and metabolome during genital infections. Sci. Rep..

[14] Yano J., Sobel J.D., Nyirjesy P., Sobel R., Williams V.L., Yu Q., Noverr M.C., Fidel P.L. (2019). Current patient perspectives of vulvovaginal candidiasis: Incidence, symptoms, management and post-treatment outcomes. BMC Women Health.

[15] Huppert J.S., Hesse E.A., Bernard M.C., Bates J.R., Gaydos C.A., Kahn J.A. (2012). Accuracy and Trust of Self-Testing for Bacterial Vaginosis. J. Adolesc. Health.

[16] Tidbury F.D., Langhart A., Weidlinger S., Stute P. (2021). Non-antibiotic treatment of bacterial vaginosis—A systematic review. Arch. Gynecol. Obstet..

[17] Angotti L.B., Lambert L.C., Soper D.E. (2007). Vaginitis: Making Sense of Over-the-Counter Treatment Options. Infect. Dis. Obstet. Gynecol..

[18] Mahan E.D., Morrow K.M., Hayes J.E. (2011). Quantitative perceptual differences among over-the-counter vaginal products using a standardized methodology: Implications for microbicide development. Contraception.

[19] Russo R., Superti F., Karadja E., De Seta F. (2019). Randomised clinical trial in women with Recurrent Vulvovaginal Candidiasis: Efficacy of probiotics and lactoferrin as maintenance treatment. Mycoses.

[20] Respecta®Balance Gel Website. https://www.giellepi.com/nutraceutical-products/respecta-md/.

[21] Yar N., Wittman E., Schaut D., De Seta F., Larsen B. (2020). Effects of Farnesol on Drug-Resistant and Non-Resistant *Candida albicans*: Implications for Cosmetic and Pharmaceutical Applications. Adv. Microbiol..

[22] Chappell B.T., Mena L.A., Maximos B., Mollan S., Culwell K., Howard B. (2021). EVO100 prevents *Chlamydia* and gonorrhea in women at high risk of infection. Am. J. Obstet. Gynecol..

[23] Moench T.R., Mumper R.J., Hoen T.E., Sun M., A Cone R. (2010). Microbicide excipients can greatly increase susceptibility to genital herpes transmission in the mouse. BMC Infect. Dis..

[24] Aroutcheva A., Gariti D., Simon M., Shott S., Faro J., Simoes J.A., Gurguis A., Faro S. (2001). Defense factors of vaginal lactobacilli. Am. J. Obstet. Gynecol..

[25] Rajan S.S., Turovskiy Y., Singh Y., Chikindas M.L., Sinko P.J. (2014). Poly(ethylene glycol) (PEG)-lactic acid nanocarrier-based degradable hydrogels for restoring the vaginal microenvironment. J. Control. Release.

[26] Owen D.H., Katz D.F. (1999). A vaginal fluid simulant. Contraception.

[27] O’Hanlon D.E., Moench T.R., Cone R.A. (2011). In vaginal fluid, bacteria associated with bacterial vaginosis can be suppressed with lactic acid but not hydrogen peroxide. BMC Infect. Dis..

[28] Wang J., Lei Y., Yu Y., Yin L., Zhang Y. (2021). Use of Acetic Acid to Partially Replace Lactic Acid for Decontamination against *Escherichia coli* O157:H7 in Fresh Produce and Mechanism of Action. Foods.

[29] Russell J.B., Diez-Gonzalez F. (1997). The Effects of Fermentation Acids on Bacterial Growth. Adv. Microb. Physiol..

[30] Graver M.A., Wade J.J. (2011). The role of acidification in the inhibition of *Neisseria gonorrhoeae* by vaginal lactobacilli during anaerobic growth. Ann. Clin. Microbiol. Antimicrob..

[31] Nardini P., Palomino R.A.N., Parolin C., Laghi L., Foschi C., Cevenini R., Vitali B., Marangoni A. (2016). *Lactobacillus crispatus* inhibits the infectivity of *Chlamydia trachomatis* elementary bodies, in vitro study. Sci. Rep..

[32] Conti C., Malacrino C., Mastromarino P. (2009). Inhibition of herpes simplex virus type 2 by vaginal lactobacilli. J. Physiol. Pharmacol..

[33] Lourenço A., Pedro N.A.A., Salazar S.B., Mira N.P. (2019). Effect of Acetic Acid and Lactic Acid at Low pH in Growth and Azole Resistance of *Candida albicans* and *Candida glabrata*. Front. Microbiol..

[34] Plummer E.L., Bradshaw C.S., Doyle M., Fairley C.K., Murray G.L., Bateson D., Masson L., Slifirski J., Tachedjian G., Vodstrcil L.A. (2021). Lactic acid-containing products for bacterial vaginosis and their impact on the vaginal microbiota: A systematic review. PLoS ONE.

[35] Hussein M.Z., Amara A.A. (2006). Case-by-case study using antibiotic-EDTA combination to control pseudomonas aeruginosa. Pak. J. Pharm. Sci..

[36] Nash E.E., Henning T.C., Pham C.D., Pettus K., Sharpe S., Kersh E.N. (2019). In vitro activity of EDTA and TOL-463 against *Neisseria gonorrhoeae*. Diagn. Microbiol. Infect. Dis..

[37] Hamoud R., Reichling J., Wink M. (2014). Synergistic antimicrobial activity of combinations of sanguinarine and EDTA with vancomycin against multidrug resistant bacteria. Drug Metab. Lett..

[38] Ghannoum M.A., Isham N., Jacobs M.R. (2011). Antimicrobial Activity of B-Lock against Bacterial and *Candida spp*. Causing Catheter-Related Bloodstream Infections. Antimicrob. Agents Chemother..

[39] Li X.-M., Wang X.-Y., Gao X.-J. (2015). Synergistic effects of lysozyme with EDTA-2Na on antibacterial activity. Beijing Da Xue Xue Bao. Yi Xue Ban.

[40] Schumacher G., Kim M., Hosseinian A., Dupon C. (1977). Immunoglobulins, proteinase inhibitors, albumin, and lysozyme in human cervical mucus: I. Communication: Hormonal profiles and cervical mucus changes--methods and results. Am. J. Obstet. Gynecol..

[41] Mitsukawa K., Otsuki K., Yanaihara A., Sawada M., Iwasaki S., Okai T. (2006). Concentration of lactoferrin and interleukin-6 in cervical mucus from patients being treated for infertility. Reprod. Med. Biol..

[42] Alakomi H.-L., Skyttä E., Saarela M., Mattila-Sandholm T., Latva-Kala K., Helander I.M. (2000). Lactic Acid Permeabilizes Gram-Negative Bacteria by Disrupting the Outer Membrane. Appl. Environ. Microbiol..

[43] Azeredo J., Azevedo N.F., Briandet R., Cerca N., Coenye T., Costa A.R., Desvaux M., Di Bonaventura G., Hébraud M., Jaglic Z. (2017). Critical review on biofilm methods. Crit. Rev. Microbiol..

[44] Lohse M.B., Gulati M., Johnson A.D., Nobile C.J. (2018). Development and regulation of single- and multi-species *Candida albicans* biofilms. Nat. Rev. Microbiol..

[45] Wu X., Zhang S., Li H., Shen L., Dong C., Sun Y., Chen H., Xu B., Zhuang W., Deighton M. (2020). Biofilm Formation of *Candida albicans* Facilitates Fungal Infiltration and Persister Cell Formation in Vaginal Candidiasis. Front. Microbiol..

[46] Naik S.T.K.K., Thangavel S., Alam A., Kumar S. (2017). Flavone Analogues as Antimicrobial Agents. Recent Pat. Inflamm. Allergy Drug Discov..

[47] Perraudin J.-P., Prieels J.-P. (1982). Lactofferrin binding to lysozyme-treated *Micrococcus luteus*. Biochim. Biophys. Acta Gen. Subj..

[48] Domínguez M.C., De La Rosa M., Borobio M.V. (2001). Application of a spectrophotometric method for the determination of post-antibiotic effect and comparison with viable counts in agar. J. Antimicrob. Chemother..

[49] Aldea M., Herrero E., Esteve M.I., Guerrero R. (1980). Surface Density of Major Outer Membrane Proteins in *Salmonella typhimurium* in Different Growth Conditions. J. Gen. Microbiol..

[50] Meur S., Sikdar A., Srivastava N., Srivastava S. (1989). Rapid photometric assay of growth of *Mycoplasma mycoides* subsp.. capri. J. Appl. Bacteriol..

[51] Ommen P., Zobek N., Meyer R.L. (2017). Quantification of biofilm biomass by staining: Non-toxic safranin can replace the popular crystal violet. J. Microbiol. Methods.

